# Circulating mature granzyme B+ T cells distinguish Crohn’s disease-associated axial spondyloarthritis from axial spondyloarthritis and Crohn’s disease

**DOI:** 10.1186/s13075-021-02531-w

**Published:** 2021-05-22

**Authors:** Adam R. Lefferts, Emilie H. Regner, Andrew Stahly, Becky O’Rourke, Mark E. Gerich, Blair P. Fennimore, Frank I. Scott, Alison E. Freeman, Ken Jones, Kristine A. Kuhn

**Affiliations:** 1 Division of Rheumatology, Department of Medicine, Anschutz Medical Campus, Aurora, CO USA; 2 Division of Gastroenterology and Hepatology, Department of Medicine, University of Colorado, Anschutz Medical Campus, Aurora, CO USA; 3 Present Address: Division of Gastroenterology, Department of Medicine, Oregon Health Sciences University, Portland, OR USA; 4 Section of Pediatric Hematology/Oncology/Bone Marrow Transplant, Department of Pediatrics, University of Colorado, Anschutz Medical Campus, Aurora, CO USA; 5 Present Address: Cascade Gastroenterology, Bend, OR USA; 6 Present Address: Department of Cell Biology, University of Oklahoma Health Sciences Center, Oklahoma City, OK USA

**Keywords:** Crohn’s disease, Inflammatory bowel disease, Axial spondyloarthritis, Ankylosing spondylitis, Interferonopathy, Cytotoxic T cells

## Abstract

**Background:**

Axial spondyloarthritis (axSpA) has strong connections with intestinal inflammation as occurs in Crohn’s disease (CD). However, the immunologic mechanisms that distinguish axSpA, CD, and those with features of both diseases (CD-axSpA) are unknown. This study aimed to address this question by initial unbiased single cell RNA-sequencing (scRNAseq) on a pilot cohort followed by validating findings using flow cytometry and ELISA in a larger cohort.

**Methods:**

Two individuals each with CD, axSpA, CD-axSpA, and healthy controls (HC) were recruited for a pilot discovery scRNAseq cohort, and the validation cohort consisted of 18 axSpA, 24 CD, 13 CD-axSpA, and 17 HC that was evaluated by flow cytometry on PBMCs and ELISAs for plasma cytokines.

**Results:**

Uniquely, PBMCs from subjects with CD-axSpA demonstrated a significant increase in granzyme B+ T cells of both CD4+ and CD8+ lineages by both scRNAseq and flow cytometry. T cell maturation was also greater in those with CD-axSpA, particularly the CD4+ granzyme B+ population. Pathway analysis suggested increased interferon response genes in all immune cell populations within CD-axSpA. Although IFN-γ was elevated in the plasma of a subset of subjects with CD-axSpA, IL-6 was also significantly elevated.

**Conclusions:**

Our findings support the presence of a chronic interferonopathy in subjects with CD-axSpA characterized by interferon signaling by pathway analysis and an expansion of mature, cytotoxic T cells. These data indicate fundamental immunological differences between CD-axSpA and both of the putative “parent” conditions, suggesting that it is a distinct disease with unique natural history and treatment needs.

**Supplementary Information:**

The online version contains supplementary material available at 10.1186/s13075-021-02531-w.

## Background

The overlapping conditions of inflammatory bowel disease (IBD) and spondyloarthritis (SpA) present unique challenges in diagnosis and treatment of individuals having both diseases. Seven percent of individuals with ulcerative colitis (UC) and 13% with Crohn’s disease (CD) will develop sacroiliitis, and 2% with UC and 4% with CD have ankylosing spondylitis (AS) [1]. Conversely, ~7% of patients with axial SpA (axSpA) will develop IBD [2], although many more patients have subclinical bowel inflammation. In spite of this overlap, clinical trials in either IBD or axSpA fail to differentiate patients with overlapping disease. Such a concept is particularly important as highlighted by trials with secukinumab, which is effective in axSpA but worsens CD [3]. While there is increased interest in identifying bowel inflammation in patients with axSpA using biomarkers such as fecal calprotectin, there is a lack of evidence to guide treatment of the overlapping IBD-associated axSpA.

In order to better treat disease, an understanding of pathophysiologic mechanisms is needed. Indeed, many groups have identified unique immune features of axSpA in the circulation, notably by IL-17-producing cell populations such as type 3 innate lymphoid cells (ILC3s) [4], mucosal-associated invariant T cells (MAIT) [5, 6], and γδ T cells [7]. In another line of work, cytotoxic granzyme-expressing CD8+ T cells in the synovial fluid uniquely associate with male AS patients [8]. However, two significant limitations exist in these studies: First, studies were based on flow cytometric analyses searching for specific cell populations; an unbiased approach for identifying altered immune phenotypes has not been performed. Second, axSpA and CD were compared to controls, lacking analysis from those with the overlapping phenotype of IBD-associated axSpA, which may demonstrate unique immune phenotypes.

To begin to address this knowledge gap, we propose an unbiased immune cell profiling using patients with IBD, axSpA, and overlapping conditions that are evaluated and compared to one another using strict definitions. In this report, we attempt such an analysis by comparing immune phenotypes in individuals with CD with ileal involvement, individuals with axSpA meeting ASAS criteria [9] with the additional requirement of positive imaging, and individuals who met the definitions of both CD and axSpA. Using these strict definitions, we began with an unbiased immune phenotyping of peripheral blood in a subset of participants. From those pilot data, we validated findings in the full cohort of recruited subjects. We find that CD-axSpA subjects, compared to CD or axSpA alone, demonstrate a change towards a Th1-like immune response with cytotoxic T cells and an interferon signature, as well as evidence of accelerated T cell maturation.

## Methods

### Subject recruitment

Utilizing a case-control format, patient and control study subjects were recruited at the University of Colorado Hospital between November 2017 and November 2018. Demographic and clinical data summarizing the study groups are presented in Table 1. Subjects were identified from the endoscopy schedule if undergoing a routine colonoscopy as part of their clinical care or recruited to undergo an elective flexible sigmoidoscopy. Recruited healthy controls (*n* = 17) were undergoing colonoscopy for routine cancer screening or a change in bowel habits. Subjects with CD (*n* = 25) were undergoing colonoscopies for disease activity assessment and colon cancer/dysplasia screening. Only patients who did not have endoscopic or histologic evidence of dysplasia were recruited into the study. AxSpA cases (*n* = 20) underwent elective flexible sigmoidoscopy for this study or were undergoing colonoscopy due to changes in bowel habits (*n* = 2) and all were evaluated for subclinical bowel inflammation; they were only included as cases when bowel inflammation by histology was excluded. Patients with CD-axSpA similarly underwent either elective flexible sigmoidoscopy (*n* = 8) or standard of care colonoscopy (*n* = 5). Subjects with CD were eligible if they had a diagnosis of such based on evaluation by an IBD-trained gastroenterologist included in the study. Subjects recruited as axSpA cases fulfilled the 2009 Assessment of SpondyloArthritis International Society (ASAS) criteria for axSpA [7], including evidence of axial disease by either MRI or radiographs. Individuals with CD-axSpA met study criteria for both CD and axSpA.

**Table 1:** Full study cohort demographics and clinical data

Condition	Controls (***N*** = 17)	CD (***N*** = 24)	axSpA (***N*** = 18)	CD-axSpA (***N*** = 13)	***p*** values*
**Age in years**	47.05 (12.3)	34.8 (14.4)	44.67 (12.1)	51.8 (11.1)	**0.0413**
**Male sex**	41.7%	41.7%	44.4%	38.5%	0.8121
**Non-Hispanic White**	88.2%	83.3%	83.3%	84.6%	0.3437
**Smoker**	23.5%	29.2%	55.6%	53.8%	0.1131
**Disease duration—months**	n/a	126 (105)	113 (127)	143 (96)	0.48
**HLA-B27 positivity**	1 (6.25%)	7 (29.2%)	14 (77.78%)	8 (61.5%)	**8.38E−05**
**TNF inhibitor usage**	0%	91.7%	88.9%	69.20%	**1.62E−09**
**SCCAI**	1.35 (1.3)	2.8 (1.9)	3.22 (1.4)	3.62 (1.5)	0.572
**Harvey Bradshaw Index**	1.3 (1.4)	2.7 (2.4)	3.1 (1.5)	3.62 (1.4)	0.778
**BASDAI**	1.66 (1.59)	2.84 (2.6)	4.9 (2.3)	5.14 (2.16)	0.53

*Significance determined using one-way ANOVA

Exclusion criteria for all groups included the presence of bowel disease (except in CD groups in which only CD was allowed), rheumatologic disease (except in the axSpA groups in which only axSpA was allowed), pregnancy, use of antibiotics in the 2 weeks prior to study entry, cancer or cancer history, inability to stop aspirin or non-steroidal anti-inflammatory drugs 7 days before and after endoscopy, use of anticoagulation, HIV, and *Clostridium difficile* infection within the past 3 months.

At the time of endoscopy, subjects completed questionnaires regarding demographic information and disease activity indices including the Harvey Bradshaw Index (HBI) and Bath Ankylosing Spondylitis Disease Activity Index (BASDAI). These data are presented in Table 1. Blood was collected into tubes containing EDTA for further processing and analysis of cells.

This study was conducted according to the principles within the Declaration of Helsinki. All study procedures were approved by the Colorado Multiple Institutional Review Board. All subjects provided written informed consent.

### Processing of PBMCs and plasma

Peripheral blood mononuclear cells (PBMCs) were isolated from collected whole blood using gradient centrifugation. Whole blood was diluted with 20 ml sterile PBS, under which 10 ml of Ficoll-Paque PLUS (GE Healthcare) was layered. After centrifugation at 300 RCF for 20 min, the buffy coat containing PBMCs was removed. PBMCs were washed with sterile PBS, counted, and cryogenically stored at a concentration of 1 × 10^6^ cells/ml in recovery freezing media (Gibco) until use.

Whole blood collected in a second K2-EDTA tube was centrifuged at 2000*g* for 5 min, and the plasma was removed and stored at −80°C. Plasma cytokines were evaluated using a multiplex plate-based platform for the cytokines IFN-α2a (lower limit of detection, LLOD= 4.0 pg/ml), IFN-β (LLOD=3.1 pg/ml), IFN-γ (LLOD=1.7 pg/ml), IL-6 (LLOD=0.33 pg/ml), IL-23 (LLOD=1.4 pg/ml), and combined IL-17A/F (LLOD=1.8 pg/ml) (Meso Scale Discovery). An additional multiplex plate evaluated cytokines TNF (LLOD=0.51 pg/ml) and IL-15 (LLOD=0.82 pg/ml).

### Single cell RNA sequencing

Cells were stained using anti-human CD45 PE (clone H130, Biolegend) and viability dye (Ghost Dye 510, Tonbo). Viable CD45+ cells were sorted on a Moflo Astrios EQ by the flow cytometry core at the University of Colorado Cancer Center. Target cell number was 5000 to 10,000 cells. Cells were submitted to the Genomics Core for sequencing using the 10x Platform (10x genomics). Recovered cell number and sequencing depth for each sample is reflected in Table 2. Sequence data was then analyzed as follows: Cellranger (2.0.2) count module was used for alignment, filtering, barcode counting, and UMI counting of the single cell FASTQs. 2D UMAP plots and clustering were determined by the following method: Seurat (V3) was used to filter cells to include only those with > 200 and < 4000 genes, mitochondrial gene expression < 10%, and total counts < 20,000. The Seurat module IntergrateData with dims = 1:20 was used to integrate the blood and colon SCRNAseq data from all 8 samples. FindClusters with resolution = 0.25 on PCA reduced data using 30 principal components was used to define clusters. UMAP coordinates were determined by Seurat. The expression of GZMB (granzyme B) and ZBTB16 (PLZF) in all cells and a subset of cells in the CD4_Tmem and CD4_Tnaive clusters were plotted using the FeaturePlot function. The average expression of GZMB and ZBTB16 in cells the CD4_Tmem cluster, separated by disease state, was calculated using the AverageExpression function. Differentially expressed genes (*p*-value < 0.05) in each cluster were determined using the FindAllMarkers function setting min.pct = 0.25 and only.pos = T. Pathway enrichment scores were generated through the use of IPA (QIAGEN Inc.). Sequencing data is publicly available in the Gene Expression Omnibus (GEO) repository under accession GSE163314.

**Table 2:** Subject information for single cell RNA sequencing

Subject details	Subject group	Sequencing run #	PBMC sequencing quality: Cell count; % saturation
51 yo Male, HLAB27−, no medications	HC	1	7399; 86.5%
36 yo Male, Crohn’s ileitis of 4 years duration, HLAB27-, moderate CD activity, on adalimumab	CD	1	6659; 86.8%
32 yo Male, AS of 7 years duration, HLAB27+, BASDAI 2.2, on infliximab	axSpA	1	6112; 88.6%
43 yo Male, AS of 10 years duration and Crohn’s ileocolitis of 5 years duration, HLAB27+, BASDAI 5.9, CD in endoscopic remission, on infliximab.	CD-axSpA	1	5001; 89.9%
51 yo Male, HLAB27−, no medications	HC	2	3737; 95.0%
33 yo Male with stricturing and internal penetrating ileal Crohn’s of 4 years duration, HLAB27−, minimal CD activity, on adalimumab and azathioprine	CD	2	11,655; 88.5%
42 yo M AS of 19 years duration, HLAB27+, BASDAI 2.2, on infliximab	axSpA	2	6041; 92.5%
45 yo M with AS of 20 years duration and newly diagnosed non-penetrating, non-stricturing Crohn’s ileocolitis of moderate severity, HLAB27+, BASDAI 1.7, on certolizumab	CD-axSpA	2	3976; 95.1%

### Quantitative PCR

Samples were thawed on ice, immediately spun down for 5 min at 10,500*g*, and washed in 1ML RPMI 1640 (Gibco) to remove DMSO. RNA was extracted using the RNeasy Plus Mini Kit (Qiagen) following the manufacturer’s recommended protocol, and cDNA was generated using the iScript cDNA synthesis kit (Bio-Rad). Quantitative PCR was performed on an Applied Biosystems 7500 real time PCR system using CYBRfast lo-ROX master mix (Tonbo). Primer sequences obtained from Primer-Bank included: *ISG15* F 5′ CGCAGATCACCCAGAAGATCG 3′ and R 5′ TTCGTCGCATTTGTCCACCA 3′; MX*1* F 5′ GTTTCCGAAGTGGACATCGCA 3′ and R 5′ CTGCACAGGTTGTTCTCAGC 3′; *IFITM1 F 5*′ CCAAGGTCCACCGTGATTAAC 3′ and R 5′ ACCAGTTCAAGAAGAGGGTGTT 3′; *IFITM3* F 5′ GGTCTTCGCTGGACACCAT 3′ and R 5′ TGTCCCTAGACTTCACGGAGTA 3′; *STAT1*F 5′ CAGCTTGACTCAAAATTCCTGGA 3′ and R 5′ TGAAGATTACGCTTGCTTTTCCT 3′; and *GAPDH* F 5′ GGAGCGAGATCCCTCCAAAAT 3′ and R 5′ GGCTGTTGTCATACTTCTCATGG 3′. Ct values for each transcript were normalized to the sample’s *GAPDH* Ct. Samples with high *GAPDH* Ct values (>30) were excluded from downstream analysis.

### Flow cytometry

Cells were stained with the indicated antibody cocktail (Supp. Tables 1 an 2) for 30 min in PBS (Gibco) with 10% FBS (Sigma) at 4°C. Following staining, cells were pelleted at 500g for 10 min and washed 3 times in 2ml PBS. Cells were fixed for 20 min (Foxp3 Transcription factor Staining kit, Tonbo). Samples were additionally stained for 30 min for intracellular markers before analysis. Samples were run on a 5-laser Becton Dickinson LSR Fortessa X-20. Analysis was carried out in FloJo V10 by Treestar. Samples with <60% viability were excluded from analysis (mean 78.8%).

### Data analysis

Data were evaluated for normality using D ’Agostino test. Normal data were evaluated by ANOVA followed by t-test with Welch’s correction and non-parametric data by Mann-Whitney. Statistical analyses and graphics were conducted with GraphPad 8.2 and Microsoft Excel Office Professional Plus 2013.

## Results

### Single cell RNA sequencing indicates mature, cytotoxic T cells, and an interferon activation signature distinguish CD-axSpA from axSpA and CD

**Fig. 1:**
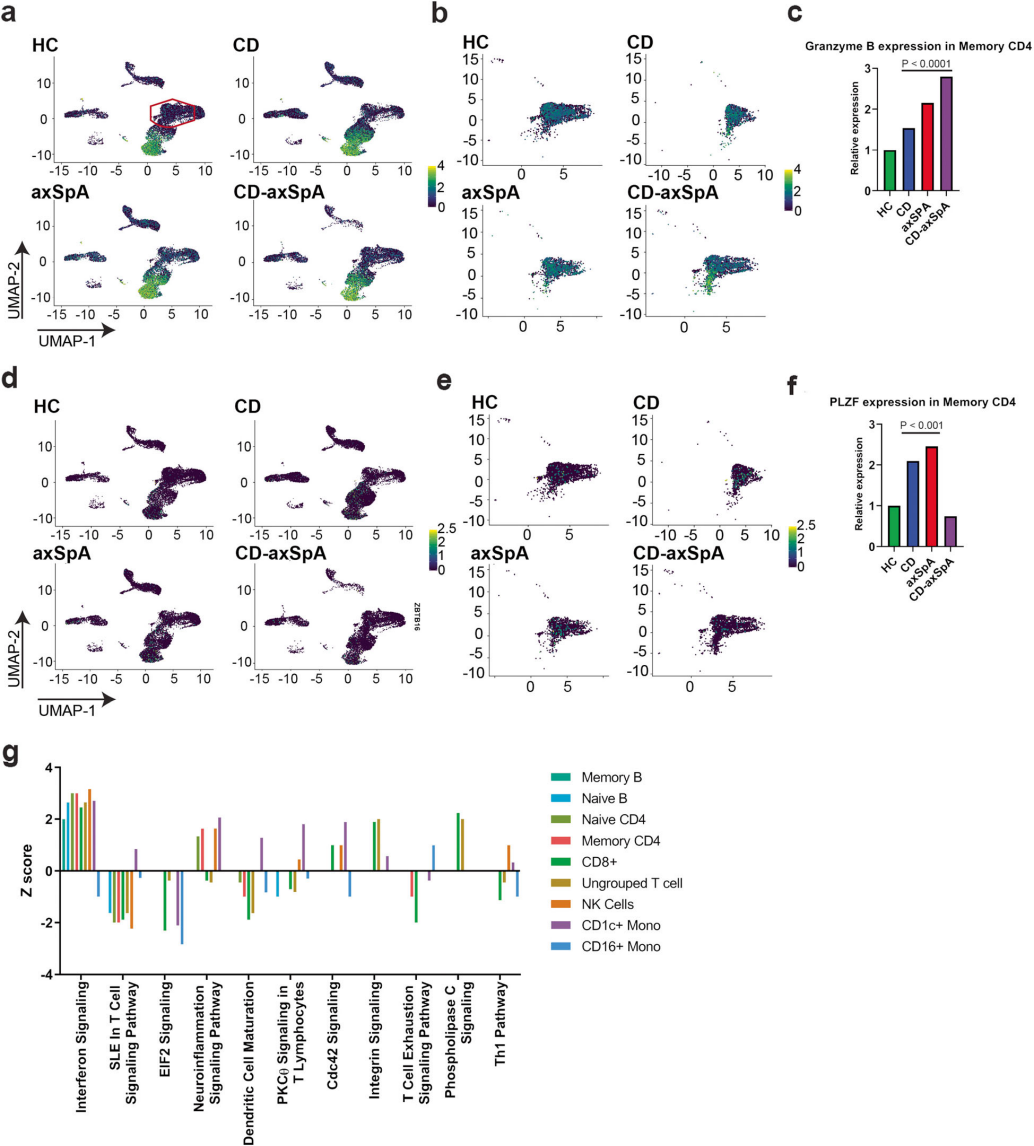
scRNAseq of PBMCs suggests an expansion of cytotoxic T cells in all disease states as well as an interferon response signature in CD-axSpA. Viable CD45+ cells sorted from PBMCs collected from 2 subjects in each group, HC, CD, axSpA, and CD-axSpA underwent scRNAseq. UMAP with (**a**, **d**) all cells, (**b**, **e**) within the memory T cell population, and (**c**, **f**) relative transcript level demonstrate (**a**–**c**) granzyme B and (**d**–**f**) PLZF expression in memory T cells across the subject groups. Intensity of expression is shown as blue (low) to moderate (green) to high (yellow) as indicated by the figure legends. The red gate in (**a**) identifies the approximate UMAP coordinates corresponding to the memory T cell population represented in (**b**, **e**). Significance as shown on the graphs was determined by Wilcoxon rank-sum test. **g** Pathway analysis of the PBMC cellular clusters from the 2 subjects with CD-axSpA was performed, and the Z-scores for pathways with at least 1 cell type with an absolute Z-score ≥ 1 is shown

To understand what cellular populations distinguish CD, axSpA, and co-morbid CD-axSpA, we first used an unbiased approach by performing high-depth single-cell RNA sequencing (scRNAseq) of the peripheral blood on a pilot cohort (Table 2). Due to the limited sample number in this application, we kept the 8 subjects as similar as possible, choosing all men, with age, disease activity, and medication use matching the larger study group. 2D UMAP analysis from the pilot cohort did not identify a uniquely expanded cell type in any disease state (Supp. Figure 1). We did, however, observe an increase in *granzyme b (GZMB*) expression within memory T cells in all disease states relative to HCs, with the highest expression in CD-axSpA (Fig. 1a–c). We additionally noted increased expression of the innate-like transcription factor promyelocytic leukemia zinc finger (*PLZF*) (Fig. 1d–f) within the memory CD4 compartment in both CD and axSpA, but not CD-axSpA, suggesting that some of the memory CD4 compartment in CD and axSpA comprised *PLZF* expressing innate-like T cells such as γδ T cells. Finally, we utilized Ingenuity Pathway Analysis (IPA) to evaluate unique activation signatures in each UMAP cellular cluster and observed an interferon signature across all cell types in CD-axSpA (Fig. 1g), and the absence of such a signature in both CD and axSpA (Supp. Figure 2).

Taking the top five transcripts of the 38 identified to drive the interferon signature by IPA (*STAT1*, *IFITM1*, *IFITM3*, *ISG15*, and *MX2*), we attempted to validate the transcriptional presence of increased IFN signaling in our larger cohort. Due to the limited PBMCs remaining from our flow cytometry analyses described next, we were able to evaluate a subset of subjects by qPCR. In total, we analyzed whole blood RNA from 14 HC (of the total 17 in the group), 21 (of 24) CD, 14 (of 18) axSpa, and 7 (of 13) CD-axSpA subjects. For the transcripts evaluated, we noted increases in *IFITM3* in the CD and axSpA groups compared to HC and a trend for increased *ISG15* transcript in CD-axSpA compared to HC (Supp. Figure 3). However, we emphasize the caveat that these data are lacking a comprehensive analysis for interferon signaling to fully validate our IPA data.

### Mature GZMB+ T cells are expanded in the circulation of subjects with CD-axSpA

**Fig. 2:**
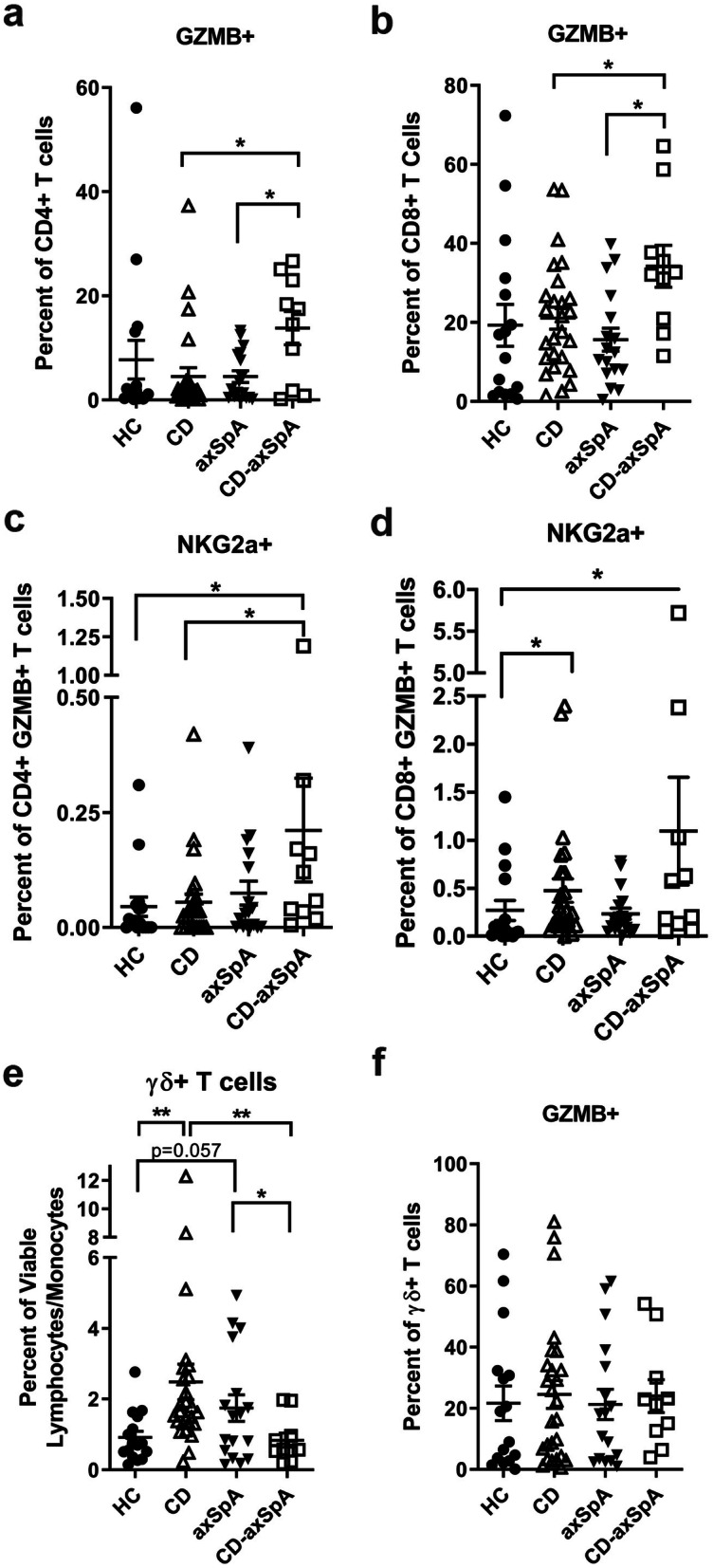
Cytotoxic GZMB+ T cells are expanded in CD-axSpA**.** Singlet, viable lymphocytes were gated for CD3+ TCRβ+ followed by CD4+ and CD8+ subsets. **a** Within the CD4+ and **b** CD8+ T cell populations, the percent GZMB+ are shown. Next, within the (**c**) CD4+ GZMB+ and (**d**) CD8+ GZMB+ T cell subpopulations, the percent expressing the inhibitory co-receptor NKG2a+ are shown. **e** Within the viable lymphocyte gate, the percent TCRγδ+ and within that population **f** the percent GZMB+ are shown. All data are individual subject values (symbols) with bars as the mean ± SEM. *, *P* < 0.05; **, *P* < 0.01 as determined by t-test for parametric data and Mann-Whitney for non-parametric data as determined by D’Agostino test

The increased *GZMB* expression in memory T cells in our scRNAseq data and the IPA analysis with a pervasive interferon response led us to probe the circulating T cell populations for the presence of cytotoxic T cells. (Consistent with the single cell data, there were no gross differences in B cells, plasma cells, pDCs, or CD11c+ mononuclear phagocytes in the peripheral blood (Supp. Figure 4a-d).) We observed a small increase in GZMB+ T cells of both CD4 (Fig. 2a) and CD8 (Fig. 2b) lineages in CD-axSpA. Within these GZMB+ subsets we also observed an increase in the NKG2a+ population within the GZMB+ sub-populations from the CD-axSpA subjects (Fig. 2c, d), which is potentially indicative of a chronically activated state [10]. We next assayed for the presence of cell surface Lamp1, a marker indicative of the recent release of cytotoxic granules [11], but did not observe significant increase in cell surface Lamp1 on CD4+ GZMB+ or CD8+ GZMB+ T cells in CD-axSpA (Supp. Figure 5a-b), suggesting that if these cells are indeed de-granulating, they are not doing so in the peripheral blood. Consistent with this observation, we did not identify increased transcriptional evidence of cellular toxicity in our scRNAseq data (data not shown), although processes of cell death like apoptosis are generally regulated at the protein level [12].

γδ TCR+ T cells were significantly expanded in the PBMCs of patients with CD compared to HC, and there was a trend towards an increase in axSpA (*P* = 0.057); however, there was no increase in subjects with CD-axSpA compared to HC (Fig. 2e) nor was GZMB expression increased on γδ T cells in any group (Fig. 2f). These findings suggest that both CD4+ and CD8+ cytotoxic T cells may contribute to the pathogenesis of CD-axSpA, while γδ T cells, which have been linked to both CD and AS [7, 13], may not be as significant in CD-axSpA.

**Fig. 3:**
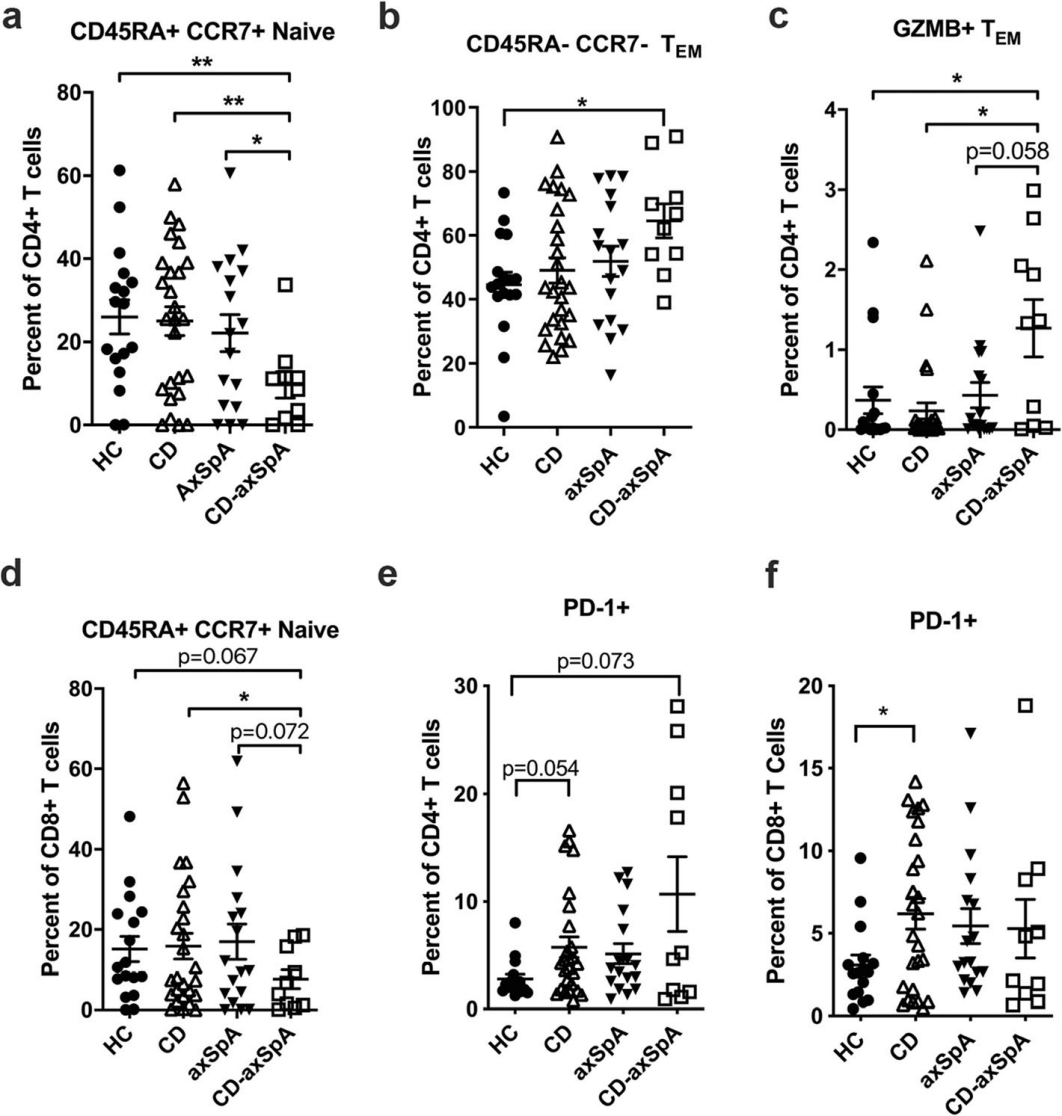
T cells in patients with CD-axSpA are skewed towards a cytotoxic effector memory profile. Singlet, viable lymphocytes gated on CD3+ TCRβ+ cells followed by CD4+ and CD8+ per group were evaluated for the percent of (**a**) CD45RA+ CCR7+ naïve cells versus (**b**) CD45RA− CCR7− effector memory T cells (T_EM_) and (**c**) GZMB+ T_EM_ within the CD4+ population. **d** Of the CD8+ T cells, the percent naïve CD45RA+ CCR7+ cells are shown. The percent of PD-1+ cells in the (**e**) CD4+ and (**f**) CD8+ populations are shown. Data are shown as individual subjects (dots) with bars as the mean ± SEM. *, *P* ≤ 0.05 as determined by Mann-Whitney for non-parametric data and t-test for parametric data as determined by D’Augostino and Shapiro-Wilk tests

The observations of increased cytotoxic T cells and interferon signaling pathway activation led us to question if the CD-axSpA patients also exhibited altered T cell maturation. PBMCs from patients with CD-axSpA had a marked decrease in circulating naïve CD45RA+ CCR7+ CD4+ T cells (Fig. 3a) as well as an increase in CD45RA− CCR7− effector memory (EM) CD4+ T cells (Fig. 3b), particularly GZMB+ T_EM_ (Fig. 3c), but not CD45RA− CCR7+ central memory (CM) T cells (Supp. Figure 6a). There was a small decrease in naïve CD8+ T cells as well (Fig. 3d), but without increases in memory CD8+ T cell subsets (Supp. Figure 6b,c). While the CD4+ compartment within CD-axSpA was skewed towards a terminally activated phenotype, we noted a small trend towards increased PD-1+ T cells of both CD4 and CD8 lineages within all disease groups including CD-axSpA (Fig. 3e, f), suggesting increased terminally exhausted T cells in at least a subset of individuals.

### CD-axSpA is distinguished from axSpA by elevated circulating IFN-y and IL-6

**Fig. 4:**
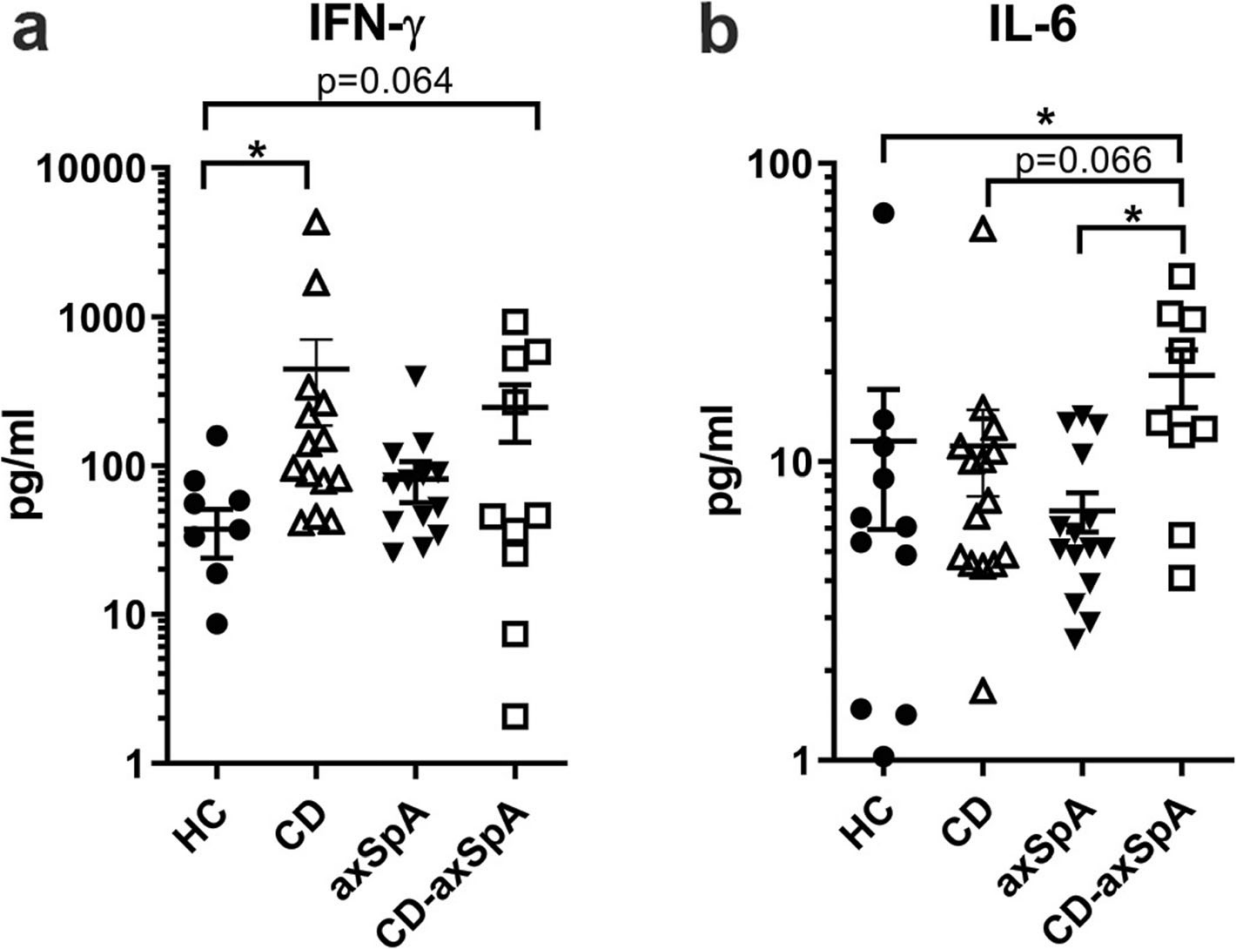
Plasma IFN-γ and IL-6 are elevated in subjects with CD-axSpA. Plasma from our cohort was evaluated for circulating cytokines by a multiplex ELISA. Symbols represent individual values of (**a**) IFN-γ and (**b**) IL-6 while bars are the group mean ± SEM in pg/ml. * denotes *P* < 0.05 determined by Mann-Whitney tests. Values below the limit of detection for the assay were not plotted

Due to the presence of an interferon signaling profile suggested by our scRNAseq IPA analysis of the peripheral blood in CD-axSpA, as well as an expansion of circulating mature cytotoxic T cells in these subjects, we assessed plasma interferons. Since we were unable to conclusively determine from IPA alone if the signaling profile was the result of type 1 (IFN-α2a and IFN-β) or type 2 interferons (IFN-γ), we chose to examine all three. Type 3 interferons do not signal in human PBMCS [14]; therefore, they were not included. We additionally assayed for Th17 pathway cytokines IL-6, IL-17A/F, and IL-23 as well as TNF given their relevance to CD and AS. Finally, we included the cytokine IL-15 as it known to upregulate GZMB in CD8+ T cells [15]. IFN-y was significantly increased in the plasma of patients with CD but only in a portion of those with CD-axSpA compared to controls (Fig. 4a), suggesting that some individuals have elevated circulating IFN-γ but other individuals with CD-axSpA derive their cellular IFN signature from signals elsewhere. There were no significant elevations in type 1 interferons in the disease groups (Supp. Figure 7a, b). We observed a significant increase in IL-6 levels in CD-axSpA relative to the other groups (Fig. 4b). IL-17A/F, IL-23, TNF, and IL-15 levels did not differ significantly among the groups (Supp. Figure 7c-f). These data suggest that the increased interferon transcriptional signature in CD-axSpA may be due to increased circulating INF-γ in only a subset of individuals, but given that IL-6 can synergize with interferons to potentiate interferon responses [16, 17], IL-6 also may be a key factor in driving the interferon signature in CD-axSpA.

## Discussion

While the clinical connection between IBD and SpA has long been appreciated, there are a lack of studies that directly compare the diseases to determine the immunologic relationship these conditions have with one another. Here, we present evidence that CD-axSpA is immunologically distinct from CD or axSpA by starting with an unbiased approach of scRNAseq to survey immune phenotypes and followed by validation through flow cytometry and ELISA. Compared to the other groups, subjects with CD-axSpA demonstrated increased circulating cytotoxic T cells of both CD4 and CD8 lineage with an overall skewing of the T cell compartment towards an activated, effector phenotype, and increased plasma IFN-y plus IL-6. These differing features between groups suggest that CD-axSpA may be a distinct disease entity from both CD and axSpA, potentially arising either as an exacerbated state of CD or axSpA characterized by new immunopathology or as a distinct disease entity with a unique natural history.

Our study is not the first to indicate cytotoxic T cells or IFN-γ in the setting of axSpA. Classically, the linkage between AS and the class I molecule HLA-B27 has suggested a CD8+ T cell pathology. Yet CD8+ T cells are significantly reduced in individuals with AS along with reduced expression of cytotoxic genes *granzyme A* (*GZMA*) and *perforin* (*PRF1*), but synovial fluid levels of GZMA and GZMB were significantly elevated compared to the peripheral blood of the same patients. Intriguingly, the differences in cytotoxic gene expression were limited to men with AS [8, 18]. In our study, we did not observe a decrease in either total CD8+ T cells or CD8+ GZMB+ T cells in the circulation of subjects with axSpA alone likely because our cohort leaned more female and was underpowered to observe sex differences. Rather, we identified a profound increase of T cells expressing GZMB in the peripheral blood of individuals with CD-axSpA, although we do not know if this expanded phenotype persists in the joints of these subjects. Intriguingly, perforin- and granzyme-expressing CD4+ and CD8+ T cells are increased in the inflamed lamina propria of patients with CD [19, 20], but, to our knowledge, such expansions have not been described in the intestine of patients with CD-axSpA or axSpA. Together with our data, these prior studies suggest a pathway in which inflamed mucosa results in the generation of cytotoxic T cells that could circulate to the joint and mediate SpA, although the specific role of these cells in disease pathology of CD-axSpA versus other disease states will need to be further investigated.

Of curiosity is the novel finding of expanded GZMB+ CD4+ TEM cells in our subjects with CD-axSpA. Cytotoxic CD4+ T cells have been shown to be potent killers of antigen presenting cells [21], and colonic epithelial cells upregulate antigen presentation in the context of IBD [22]; it is possible that the cytotoxic CD4 cells observed in CD-axSpA directly mediate bowel disease through cytolytic killing of epithelial cells. Equally, these cells may have targets within the joint space, and/or influence pathology through the direct proteolytic effects of granular proteins. Therefore, elucidating the potential role of cytotoxic T cells in the joint will require an understanding of the potential cellular targets, with the caveat that they may have little or no meaningful impact in disease pathogenesis, and their generation is simply a side effect of the presence of elevated systemic interferon.

Our findings point towards IFN-γ being a key driver of CD-axSpA, since cytotoxic T cells of both CD4 and CD8 lineages respond to IFN-y [23, 24], and we observed a profound IFN response gene signature by IPA. The loss of circulating naïve T cells in our subjects with CD-axSpA may also be a consequence of a chronic interferon response, as this has been observed in other conditions characterized by chronic interferon exposure such as Down’s syndrome and hepatitis C infection [25, 26]. Our study, though, does not address the source(s) of the elevated IFN-γ and IL-6 observed in CD-axSpA, and it remains unclear whether these cytokines are primary drivers of, or a consequence following from, the underlying pathology.

Our data are in partial agreement with Smith et al. who previously identified increased IFN-γ responses in ex vivo derived macrophages from circulating monocytes in individuals with AS [27]. The derived macrophages from AS versus healthy controls demonstrated an elevated IFN-γ responsive gene signature although they expressed less IFN-γ; however, when stimulated with IFN-γ, the derived macrophages did not upregulate IFN-γ response genes like healthy controls, suggesting an intrinsic defect in the IFN-γ pathway in patients with AS [27]. We did not observe the same IFN-γ response in monocytes from axSpA subjects, but rather in our CD-axSpA subjects, in our scRNAseq dataset. Such discrepancies may be due to limited numbers in our scRNAseq analysis, our more restrictive subsetting of axSpA with and without concomitant CD, or the effect of ex vivo differentiation in the Smith et al. study [27].

IFN-γ also has been linked to IBD primarily acting through weakening of epithelial junctions in the intestine [28]. Thus, it seems likely that IFN-γ acts as a driver of the intestinal pathology observed in CD-axSpA. IL-6 has also been linked to disease activity in IBD [29]. Furthermore, IFN-γ may be involved in the joint disease observed in CD-axSpA, as it has the potential to disrupt osteoclast differentiation through suppression of the RANK signaling pathway [30]. IL-6, by a distinct mechanism involving suppression of the NF-κB and JNK pathways, is also known to suppress osteoclast differentiation [31]. As such, IFN-γ and IL-6 may be able to mediate joint damage through different mechanisms than the classically IL-17-driven processes observed in axSpA [32]. Additionally, the synergistic effects of concurrent IL-6 and IFN-γ signaling [16, 17], which amplifies IFN-γ responses through IL-6 mediated IRF1 and IRF9 expression, may contribute to the unique pathology observed in CD-axSpA and explain why there was an interferon response signature in CD-axSpA and not CD, despite similar plasma IFN-γ levels.

We did identify a second transcript by scRNAseq—*PLZF*—that was significantly elevated in CD and axSpA, but not CD-axSpA compared to controls. PLZF is a transcription factor that regulates the development of innate-like T cells including γδ T cells, NK T cells, MAIT cells, and innate lymphoid cells (ILCs) [33–36]. Emerging data suggest that PLZF may also be important in fetal T cell development, particularly those effector CD4+ T cells from the gut [37]. Certainly, increases in PLZF-mediated populations like ILC3s, NK T cells, γδ T cells, and subsets of MAIT cells are observed in the circulation of subjects with axSpA [4–7, 38]. While we did observe increases in γδ T cells in the circulation from subjects with CD and axSpA, consistent with previous reports [7, 13], we did not evaluate NK T or MAIT cell populations nor fully validate the increased expression of *PLZF* in CD and axSpA. However, PLZF in SpA specifically has only been evaluated, to our knowledge, in the peripheral blood and synovial fluid invariant NK T and γδ T cells. Compared to peripheral blood invariant NK T and γδ T cells from controls, the same cellular populations in the peripheral blood and synovial fluid of subjects with SpA did not have significant changes in PLZF protein [38]. Therefore, the role for PLZF in SpA, and more specifically, differentiating CD-axSpA remains unclear.

Unfortunately, this study is underpowered to determine the answers to a number of additional critical questions regarding CD-axSpA. For example, distinctions between B27+ and B27− presentations of both axSpA and CD-axSpA may be missed as we included only B27+ individuals in our discovery cohort. Having a larger discovery cohort would have allowed for the discernment of more subtle phenotypes. Additionally, usage of TNF inhibitors within our study cohort makes comparisons between each disease state and healthy controls more difficult. We chose to utilize patients under treatment due to the lack of new diagnosis CD-axSpA patients, as in our cohort patients were invariably under treatment for either CD or axSpA by the time of a diagnosis of CD-axSpA. However, our cohort is well matched for TNF inhibitor usage between disease states, making internal comparisons within diseases important as each group responds differently to treatment, highlighting underlying immunological differences between CD, axSpA, and CD-axSpA. Ideally, patient samples would be collected at the time of diagnosis to remove the confounding effects of treatment; however, due to the unique features of CD-axSpA, a prospective cohort will be required to more fully explore the immunological basis of this disease.

## Conclusions

This study provides evidence, by cytokine signature and cellular immunophenotype, that CD-axSpA may be best thought of as a distinct disease entity, instead of an exaggerated form of either CD or axSpA. We caution that the data are limited to a highly defined clinical phenotype and may not explain other forms of IBD or peripheral SpA in conjunction with IBD. Yet our findings highlight the need for studies in well-phenotyped populations in order to understand the constellation of diseases under the SpA and IBD umbrellas that are likely immunologically distinct entities. Such studies can then shed light on how best to treat these complex patients.

## Supplementary Information

**Additional file 1.**

## Data Availability

All data are available upon request. Sequencing data is publicly available in the Gene Expression Omnibus (GEO) repository under accession GSE163314.

## Abbreviations

IBD: Inflammatory bowel disease
CD: Crohn’s disease
UC: Ulcerative colitis
AS: Ankylosing spondylitis
SpA: Spondyloarthritis
axSpA: Axial spondyloarthritis
CD-axSpA: Crohn’s disease-associated axial spondyloarthritis
scRNAseq: Single cell RNA sequencing
PBMC: Peripheral blood mononuclear cells
ASAS: Assessment of SpondyloArthritis International Society
BASDAI: Bath Ankylosing Spondylitis Disease Activity Index
HBI: Harvey Bradshaw Index
*GZMB*: *Granzyme b*
*PLZF*: Innate-like transcription factor *promyelocytic leukemia zinc finger*
Th1: Type 1 helper T cells
Th17: Type 17 helper T cells
TEM: Effector memory T cells
TCM: Central memory T cells
