# Supplementary material for: Circulating mature granzyme B+ T cells distinguish Crohn’s disease-associated axial spondyloarthritis from axial spondyloarthritis and Crohn’s disease
Source: Arthritis Res Ther. 2021 May 22;23:147. doi: 10.1186/s13075-021-02531-w (PMC8140495; doi:10.1186/s13075-021-02531-w)
Supplement: Supplementary file 1 — Additional file 1. [file 13075_2021_2531_MOESM1_ESM.pdf]

Supplemental Figure 1

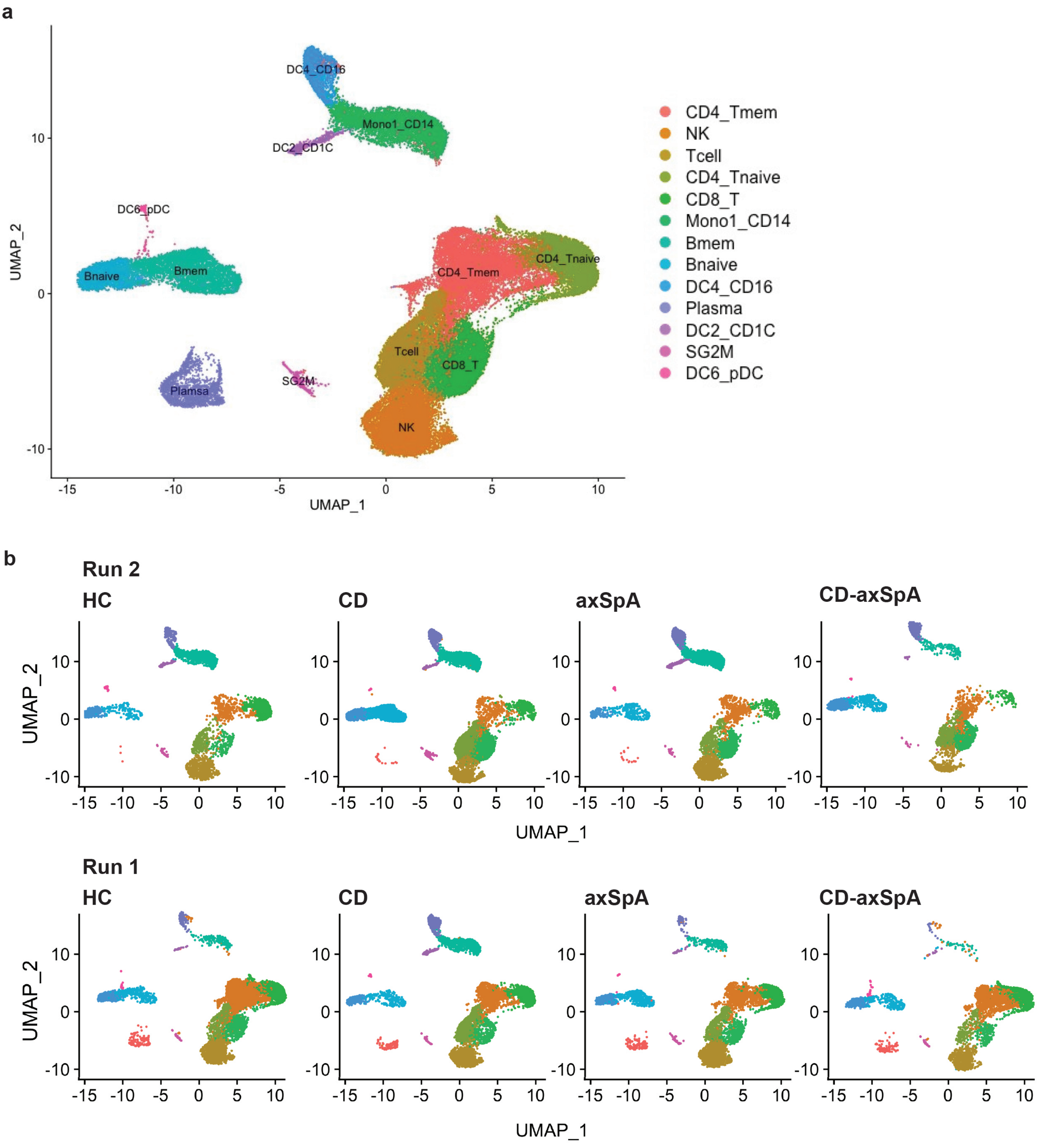

Supplemental Figure 1. UMAP analysis does not identify uniquely enriched cellular clusters across disease states. (a) UMAP results colored by putative cell type. (b) Results broken down by subject do not reveal enrichment for any particular cluster in any disease state.

Supplemental Figure 2

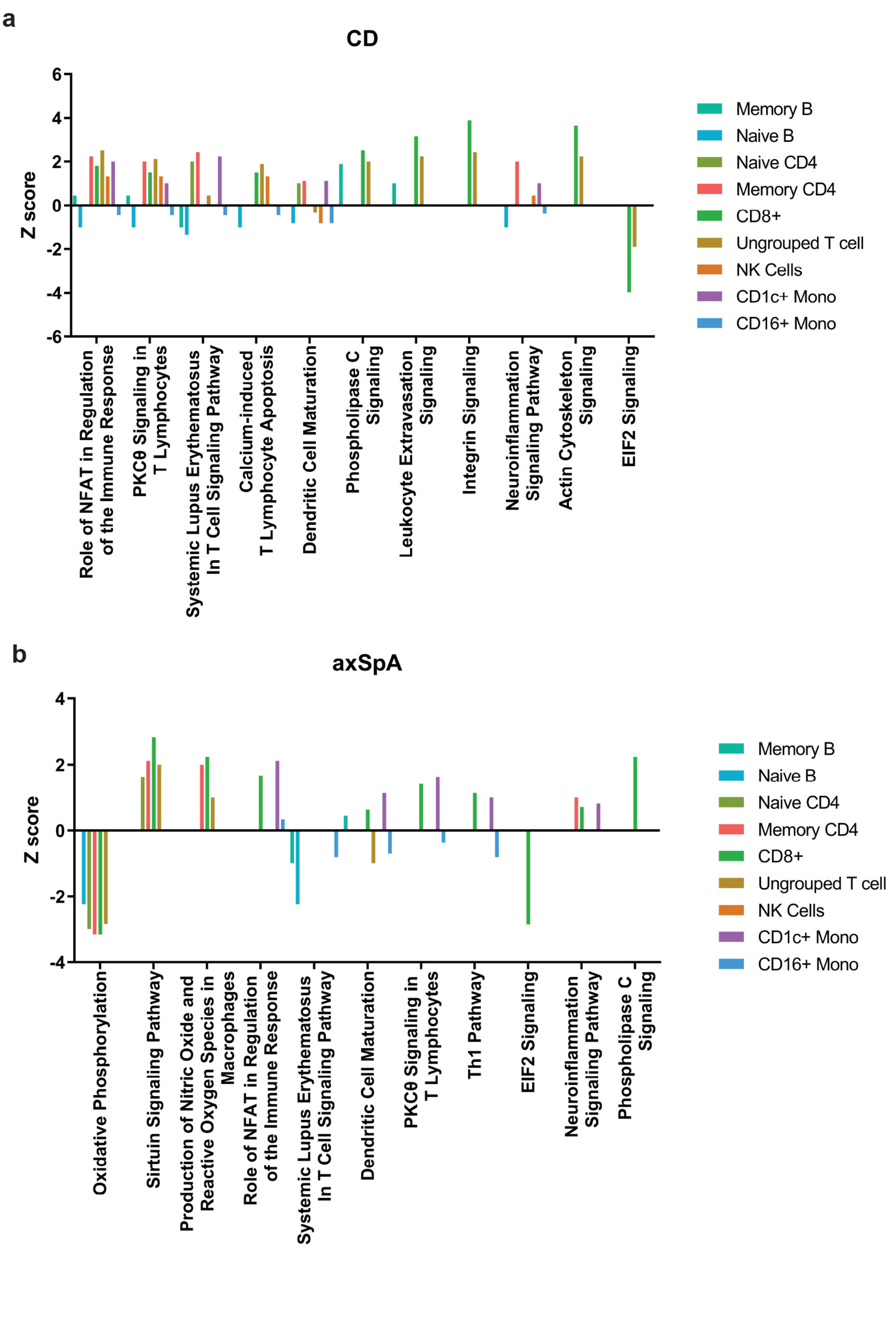

Supplemental Figure 2. An IFN response is absent in pathway analysis of single cell RNA expression in PMBCs of subjects with CD and axSpA. IPA of RNA expression in the PBMCs from the 2 subjects with (a) CD and (b) axSpA was performed. The Z-scores for pathways with at least 1 cell type with an absolute Z-score ≥ 1 is shown.

Supplemental Figure 3

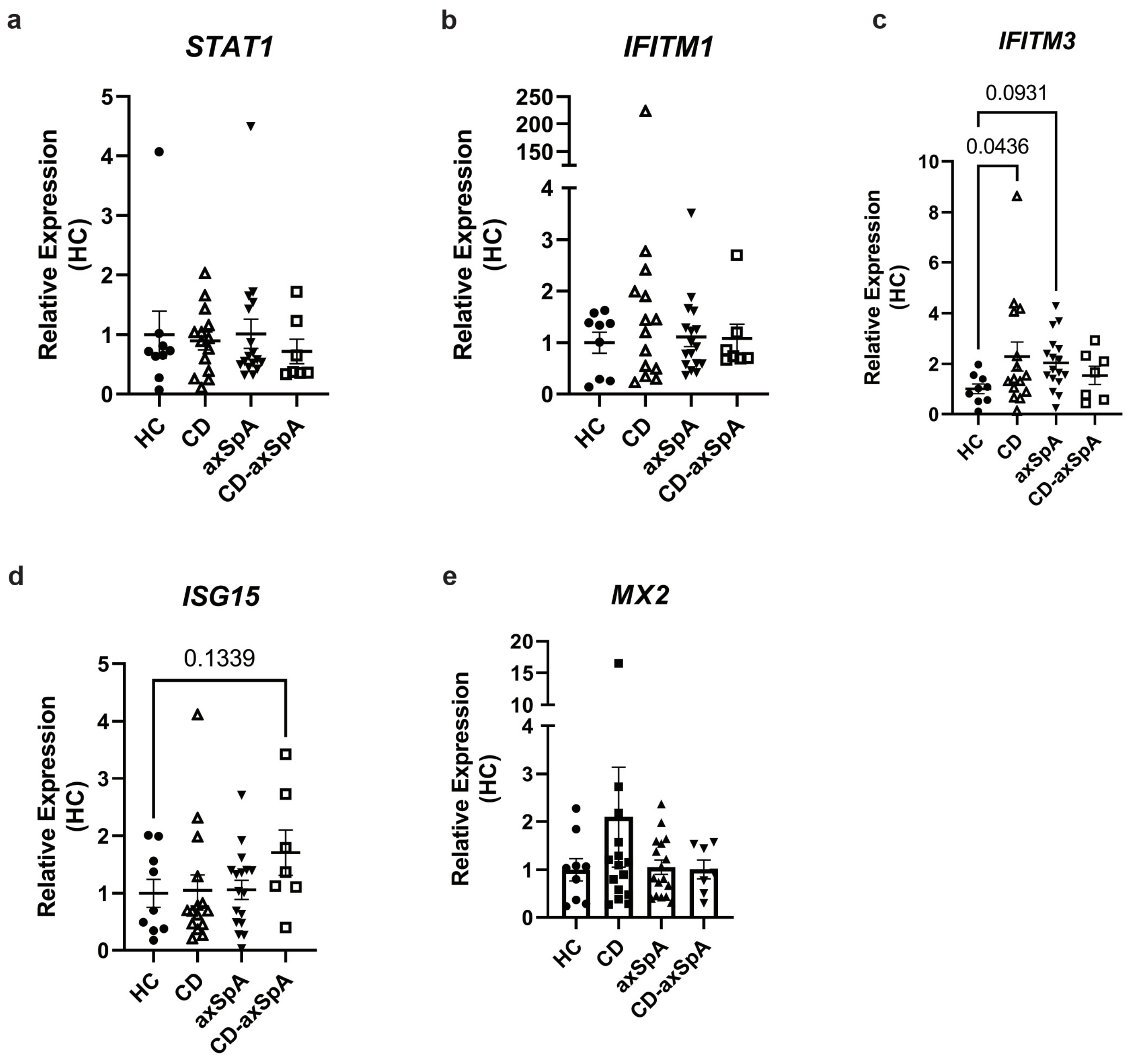

Supplemental Figure 3. Five of the 38 transcripts tested for interferon signaling demonstrate minimal changes between subject groups. Whole blood RNA extracted from at least 1 x 10^6 PBMCs in each subject was evaluated by qPCR for the expression of (a) STAT1, (b) IFITM1, (c) IFITM3, (d) ISG15, and (e) MX2. Each gene was normalized to GAPDH and shown as the relative expression compared to HC for each subject (dots) and group mean ± SEM (bars). Significance was evaluated using an unpaired t-test and p-values noted.

Supplemental Figure 4

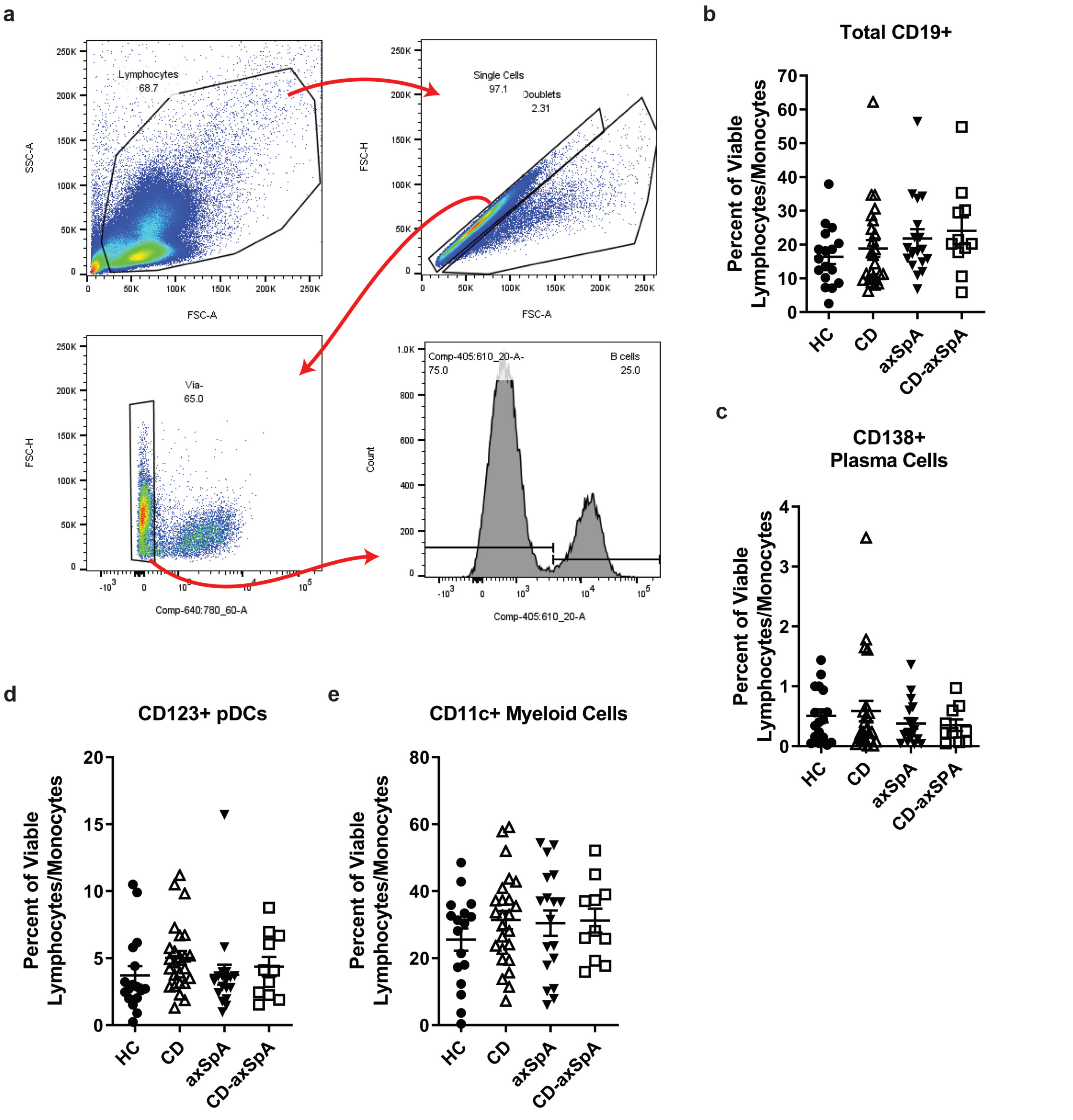

Supplemental Figure 4. Gross percentages of B cells and myeloid lineage cells do not differ significantly between disease states. (a) Flow cytometry gating of lymphocytes+monocytes based on forward (FSC) and side (SSC) scatter followed by singlets and then viable cells gated for lineage markers such as CD19. Percent of viable lymphocytes/monocytes that are (c) total CD19+ B cells, (c) CD19+ CD138+ plasma cells, (d) CD123+ plasmacydoid dentritic cells, and (e) CD11c+ myelioid cells are quantified in each subject. Data are individual subjects (dots) and group mean ± SEM (bars).Populations were not significantly different as determined by one-way ANOVA.

Supplemental Figure 5

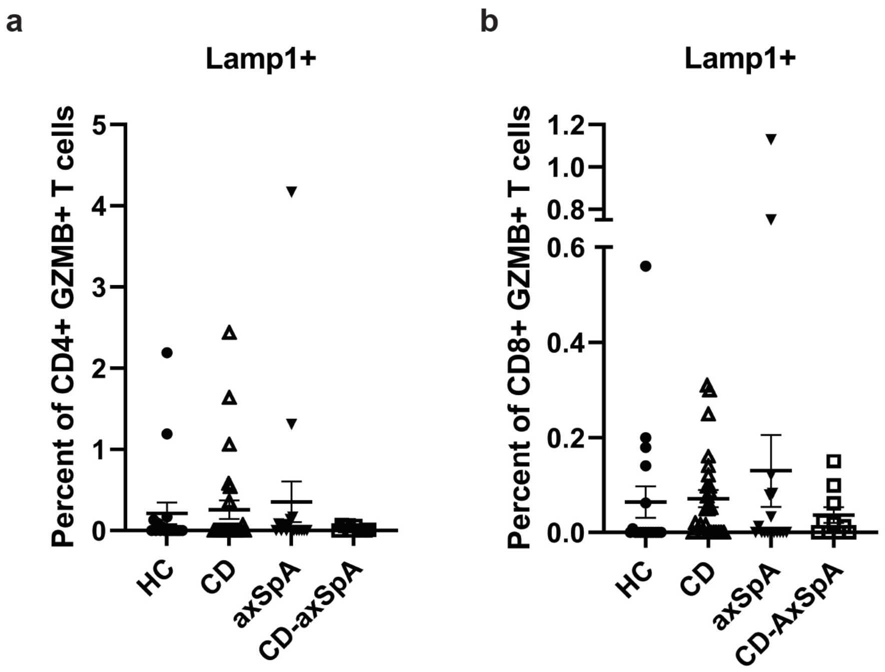

Supplemental Figure 5. Lamp1 expression does not differ on GZMB+ T cells across study groups.
By flow cytometry, singlet viable lymphocytes were gated for CD3+ TCRβ+ cells. Within that T cell population, (a) CD4+ GZMB+ or (b) CD8+ GZMB+ populations were evaluted for the presence of Lamp1. By ANOVA, no statistically significant differences were found between study groups.

Supplemental Figure 6

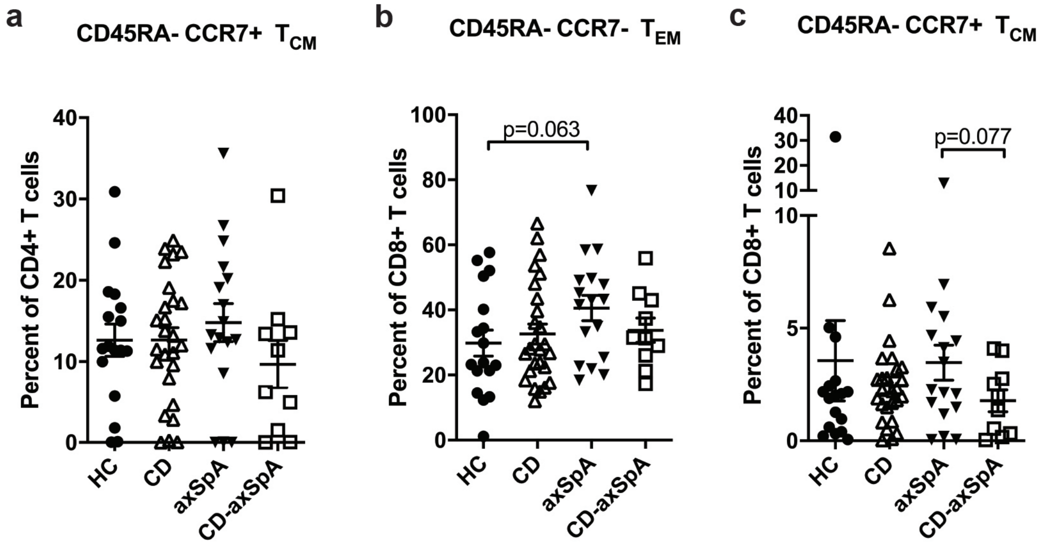

Supplemental Figure 6. T cell memory subsets in CD4+ and CD8+ subpopulations. By flow cytometry, singlet, viable lymphocytes were gated for CD3+ TCRβ+ cells and then CD4+ or CD8+. Within the CD4+ and CD8+ T cell populations, (a) CD4+ CD45RA- CCR7+ central memory TCM cells, (b) CD8+ CD45RA- CCR7- effector memory TEM cells, and (c) CD8+ CD45RA- CCR7+ TCM cells were evaluted. Data are shown as individual subject values (dots) and bars as the mean pg/ml ± SEM for each group. P-values were determined by unpaired t-tests.

Supplemental Figure 7

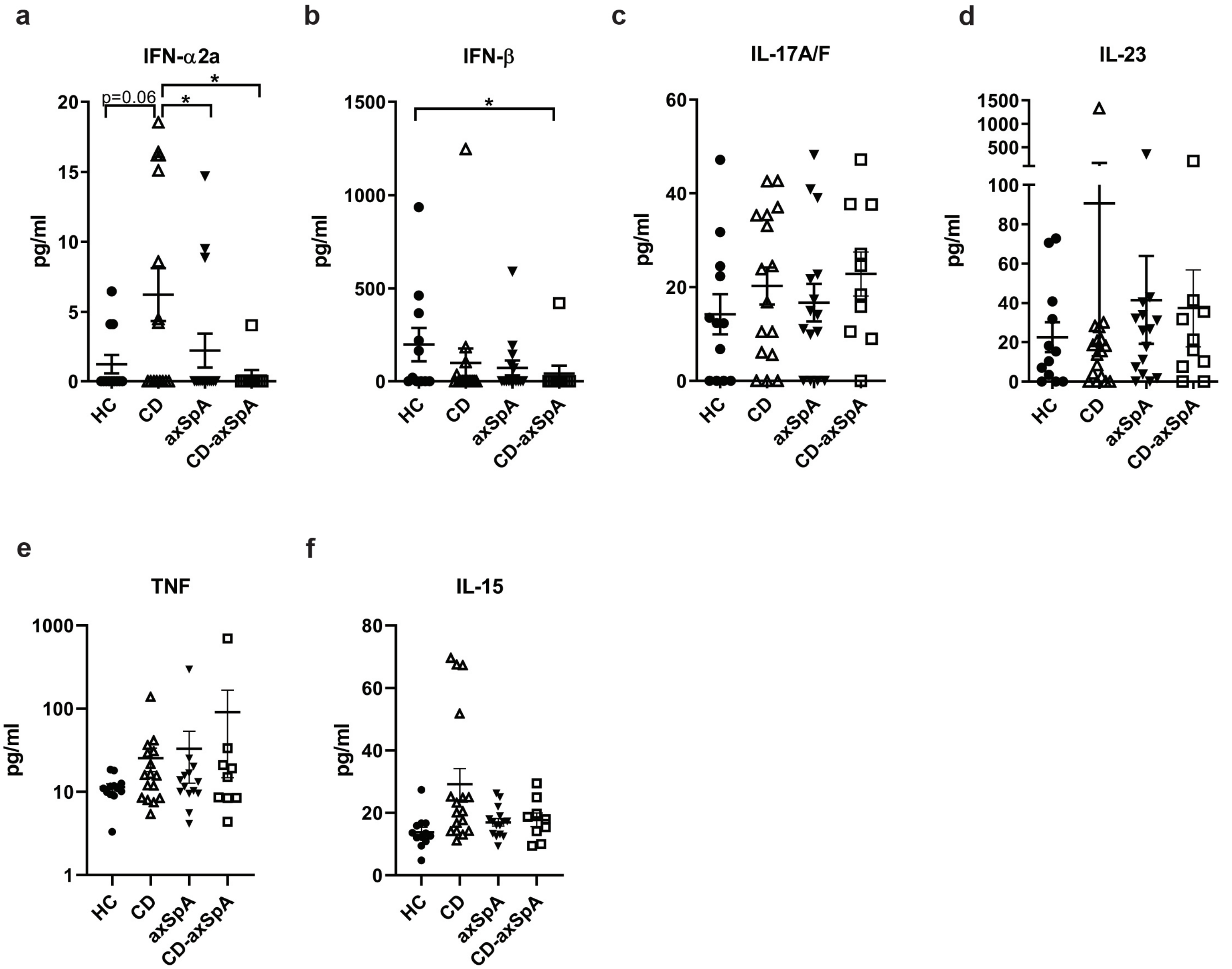

Supplemental Figure 7. Plasma type-1 interferons and interleukins 17 and 23 do not differentiate disease states. Plasma from subjects with CD, axSpA, and CD-axSpA as well as HC were assayed for cytokines using a multiplex ELISA. Type I interferons (a) IFN-α2a and (b) IFN-β as well as (c) IL-17A/F, (d) IL-23, (e) TNF, and (f) IL-15 are shown as individual subject values (dots) and bars as the mean pg/ml ± SEM for each group. No significant differences between groups was determined by Mann-Whitney.

Supplemental Table 1. Flow cytometry antibody staining panel #1 for PBMCs

**Specificity**	**Clone**	**Flourophore**	**Company**
CD56	B159	BB515	BD
CD123	7G3	BB700	BD
VA 7.2	OF-5A12	PE	BD
CD1A	HI149	APC	BD
GRANZYME B	GB11	AF700	BD
Fixable viability Stain	N/A	780	BD
CD138	MI15	BV421	BD
CD19	SJ25C1	BV605	BD
CD161	DX12	BV711	BD
CD11C	B-ly6	BV786	BD
CD3	SK7	BUV395	BD

Supplemental Table 2. Flow cytometry antibody staining panel #2 for PBMCs

**Specificity**	**Clone**	**Flourophore**	**Company**
CD8	RPA-T8	BB515	BD
Tcr gd	11F2	BB700	BD
CD107a	H4A3	PE	BD
CCR7	150503	PE-CF594	BD
CD27	M-T271	PE-cy7	BD
Tcr ab	T10B9.1A-31	APC	BD
Granzyme B	GB11	AF700	BD
Fixable viability Stain	N/A	780	BD
NKG2a	131411	BV421	BD
PD-1	EH12.1	BV480	BD
CD19	SJ25C1	BV605	BD
CD28	CD28.2	BV711	BD
CD11c	B-ly6	BV786	BD
CD4	RPA-T4	BUV395	BD
CD45RA	HI100	BUV737	BD
